# Functionally dissociating temporal and motor components of response preparation in left intraparietal sulcus

**DOI:** 10.1016/j.neuroimage.2010.09.038

**Published:** 2011-01-15

**Authors:** Julien Cotti, Gustavo Rohenkohl, Mark Stokes, Anna C. Nobre, Jennifer T. Coull

**Affiliations:** a Laboratoire de Neurobiologie de la Cognition, Université de Provence & CNRS, Pôle 3C, 3 Place Victor Hugo, 13331 Marseille Cedex 3, France; b Department of Experimental Psychology, University of Oxford, South Parks Road, Oxford OX1 3UD, UK

**Keywords:** Motor attention, Motor preparation, Motor intention, Temporal preparation, Supramarginal gyrus

## Abstract

To optimise speed and accuracy of motor behaviour, we can prepare not only the type of movement to be made but also the time at which it will be executed. Previous cued reaction-time paradigms have shown that anticipating the moment in time at which this response will be made (“temporal orienting”) or selectively preparing the motor effector with which an imminent response will be made (motor intention or “motor orienting”) recruits similar regions of left intraparietal sulcus (IPS), raising the possibility that these two preparatory processes are inextricably co-activated. We used a factorial design to independently cue motor and temporal components of response preparation within the same experimental paradigm. By differentially cueing either ocular or manual response systems, rather than spatially lateralised responses within just one of these systems, potential spatial confounds were removed. We demonstrated that temporal and motor orienting were behaviourally dissociable, each capable of improving performance alone. Crucially, fMRI data revealed that temporal orienting activated the left IPS even if the motor effector that would be used to execute the response was unpredictable. Moreover, temporal orienting activated left IPS whether the target required a saccadic or manual response, and whether this response was left- or right-sided, thus confirming the ubiquity of left IPS activation for temporal orienting. Finally, a small region of left IPS was also activated by motor orienting for manual, though not saccadic, responses. Despite their functional independence therefore, temporal orienting and manual motor orienting nevertheless engage partially overlapping regions of left IPS, possibly reflecting their shared ontogenetic roots.

## Research Highlights

►Anticipating when and how to respond can be functionally and neurally dissociated. ►Temporal orienting (when) engages left IPS for both oculomotor and manual responses. ►Motor orienting (how) engages left IPS only for manual, not oculomotor, responses. ►Manual action circuits may be functionally recycled for anticipating *when* to respond.

## Introduction

A soccer player receiving a pass uses information contained within the ball's trajectory to prepare both when the ball will reach him and with which part of his body (foot, chest, head) he will receive the pass. In the laboratory, it has repeatedly been shown that directing (or “orienting”) attention towards the expected spatial location (Posner et al., 1980) or temporal onset (Niemi and Näätänen, 1981; Nobre, 2001) of an upcoming event (e.g., the ball's arrival) optimises responses to that event, as does selectively attending to the motor effector expected to execute the response (Rosenbaum, 1985; Rushworth et al., 2003).

While spatial orienting of attention has long been linked to right parietal cortex (Corbetta et al., 1993; Mesulam, 1981), both temporal (Coull and Nobre, 1998) and motor (Rushworth et al., 2003; Hesse et al., 2006) orienting of attention recruit similar regions of left parietal cortex. Neuroanatomical overlap in fronto-parietal circuits for spatial orienting and saccade preparation (e.g., Corbetta et al., 1998; Nobre et al., 2000) has been used to support the premotor theory of attention (Rizzolatti et al., 1994), which posits that (oculo)motor preparation for a spatially defined action guides the deployment of attentional resources in space. By analogy, preparation for a delayed action may also guide appropriate deployment of attentional resources in time. If so, the aforementioned neuroanatomical overlap in left parietal cortex for temporal and motor orienting could reflect their functional overlap. For example, preparation for action may induce a concomitant expectation of when that action is likely to be executed (Requin et al., 1991). Conversely (though not mutually exclusively), temporal expectation of an event's onset may automatically invoke (or “afford”) preparation of a motor effector suitable for responding to that event (Gibson, 1979). We set out to determine whether it is possible to dissociate motor and temporal orienting, from both behavioural and neuroanatomical points of view. We predicted temporal orienting would benefit performance, and recruit left parietal cortex, even when the motor effector required to register the response was unpredictable.

Prior fMRI studies have used hand movements to measure performance benefits of temporal and motor orienting (Coull and Nobre, 1998; Rushworth et al., 2003) or predictability (Sakai et al., 2000). To test whether activation of left parietal cortex in these studies was specific to preparation of manual responses, we also examined temporal and motor orienting for saccadic eye movements. Prior behavioural studies have confirmed that temporal predictability can speed both smooth pursuit (de Hemptinne et al., 2007; Jarrett and Barnes, 2005) and saccadic eye movements (Bronstein and Kennard, 1987). Our mixed fMRI design allowed us to compare and contrast behavioural and neural correlates of temporal and motor orienting on hand versus eye movements within the same experimental paradigm. Moreover, we deliberately disentangled motor preparation from potential spatial confounds by using endogenous cues that did not allow preparation of a spatially selective response (e.g., left/right hand) but, rather, selective preparation of an entire effector system (ocular/manual) (see also Dickinson et al., 2003; Beurze et al., 2009). Both motor and temporal orienting could therefore be investigated independently of spatial orienting influences.

Finally, it should be noted that we have coined the terms “motor orienting” and “temporal orienting” deliberately in order to provide a direct analogy with the process of spatial orienting. With spatial orienting, attentional resources are directed to a specific location in space, with temporal orienting they are directed to a specific moment in time, while with motor orienting they are directed to a specific motor effector. The term motor orienting is thus equivalent to a selective form of motor preparation or motor intention. However, we prefer to retain our “orienting” terminology in order to make clear that similar attentional mechanisms can operate across different processing domains.

## Methods

A behavioural experiment was conducted before the scanning session to establish the validity of the experimental design, i.e., to evaluate the capacity of symbolic cues to induce attentional orienting independently along the motor and temporal dimensions.

### Behavioural experiment

#### Subjects

Fifteen subjects took part in the experiment (mean age 26 years; 6 females). All were healthy, with normal or corrected-to-normal vision. They were naïve as to the purpose of the study and gave informed consent. The study was performed in accordance with the ethical standards laid down in the Declaration of Helsinki (last modified 2004).

#### Apparatus

Subjects were seated in complete darkness on an ergonomic posture chair, with their head maintained straight ahead by a chin rest and a frontal support. They faced a 19″ LCD screen that presented visual stimuli at 60 Hz. A helmet-mounted infra-red sensor allowed recording of left eye position at 500 Hz (EyeLink II infra-red eye tracking system, SR Research, Mississauga, Ontario, Canada) with a spatial resolution of > 0.1°. Precise measurements of horizontal and vertical eye position were achieved with a nine-point calibration grid. Saccade onsets were determined offline using data analysis programs implemented in Matlab (MathWorks, Natick, MA; multi-criterion detection using thresholds of velocity: 15 deg s^− 1^, acceleration: 3000 deg s^− 2^, and horizontal displacement: 1.5 deg). Button responses were recorded using a laboratory-made response box with two low trigger-force buttons that sampled manual responses at 1000 Hz. A real-time acquisition system (Keithley Instruments, Cleveland, OH) controlled the experiments using laboratory-made software (Docometre).

#### Experimental procedure

**Fig. 1:**
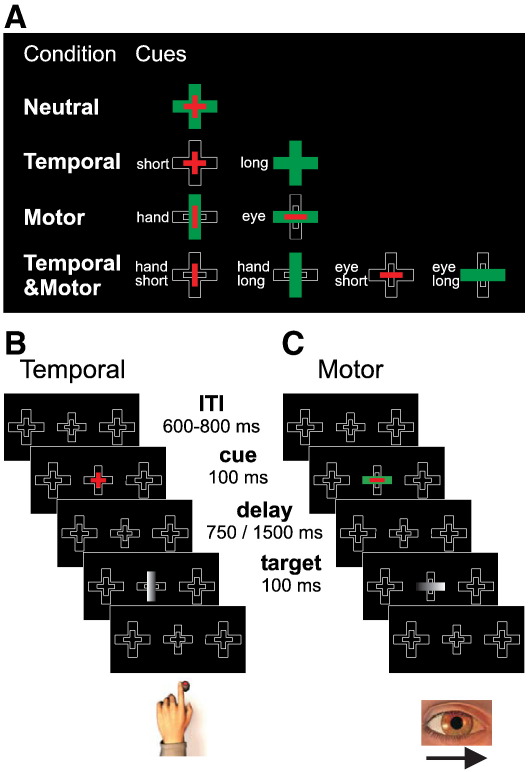
Sequence of events in a trial. After a random inter-trial interval (ITI), a cue was presented centrally for 100 ms. After one of two possible delays (750/1500 ms), a target was presented centrally for 100 ms. The motor response to be given was indicated by the orientation of the target, with vertical targets specifying button-presses and horizontal targets specifying saccades. The laterality of the response was determined by target shading, such that subjects made left-/right-hand button-presses or left/rightward saccades to the lighter side of the target. A) The presence (+) or absence (−) of temporal (T) or motor (M) orienting cues were crossed in a 2 × 2 factorial design, yielding four cueing conditions: Temporal (T+M−), Motor (T−M+), Temporal&Motor (T+M+), Neutral (T−M−). Each of these conditions was associated with a specific set of centrally presented cues, which are illustrated here. B) Illustrative example of a trial in the ‘Temporal’ condition. The cue informed the subject that the target would appear after a short delay. After a short delay, the appearance of a vertical target with the lighter side on the left instructed the subject to produce a leftward manual response. C) Illustrative example of a trial in the ‘Motor’ condition. The cue informed the subject that the response to be produced would be a saccade. After a variable delay, the appearance of a horizontal target with the lighter side on the right instructed the subject to produce a rightward saccadic response. These are simply illustrative examples: all combinations of cue-type/delay/effector/side were presented.

Subjects performed a choice-RT task. A background scene, comprising one central and two peripheral compound crosses (located ± 10 deg horizontally from screen centre), was present throughout the task (Figs. 1B & C). The central cross acted as a place-marker for the presentation of cues and targets, while the peripheral crosses acted as response zones for lateral eye movements. Every trial had the same sequence of events. Trials started with central fixation for a variable period (600 ~ 800 ms). A visual cue was presented centrally for 100 ms. After one of two possible delays (ISI: 750 ms/1500 ms) a visual target was then presented centrally for 100 ms. Subjects were required to react to it as quickly as possible using one of two possible movements (hand/eye), which was indicated by the orientation of the target. A vertical target specified a hand movement (manual button-press) whereas a horizontal target specified an eye movement (oculomotor saccade). The laterality (left/right) of the response was determined by the horizontal black-to-white grating of the target, with subjects being required to respond on the side corresponding to the lighter side of the target. Therefore, subjects pressed a button placed under the index finger of the hand corresponding to the lighter side of vertical targets (for example see Fig. 1B) or produced a rapid eye movement (saccade) toward the response zone located on the lighter side of horizontal targets (for example see Fig. 1C). In all four cueing conditions (see below), the motor effector used to produce the response (eye/hand) *and* the time between cue and target presentation (750 ms/1500 ms) varied on a trial-by-trial basis. All four conditions were therefore matched for sensori-motor requirements and trial timing, with the only difference between conditions being the attentional set of the subject.

The task was designed to manipulate subjects' expectations of when or with which motor effector a response would be made. A 2 × 2 factorial design, comprising the factors of motor effector and temporal cueing, yielded four cueing conditions (Fig. 1A):

1. In the ‘Motor’ orienting condition, the cue indicated which motor effector should be used to respond (eye/hand), with saccadic and manual trial-types varying on a trial-by-trial basis. No information was provided concerning the time between cue and target, which varied (short/long) in a counterbalanced manner across saccadic and manual trial-types.
2. In the ‘Temporal’ orienting condition, the cue provided predictive information concerning the time between cue and target (750 ms/1500 ms), with short and long trial-types varying on a trial-by-trial basis. No information was provided concerning the motor effector that should be used to produce the response, which varied (eye/hand) in a counterbalanced manner across short and long trial-types.
3. In the ‘Temporal&Motor’ orienting condition, the cue indicated both the time between cue and target (750 ms/1500 ms) and which motor effector should be used to make the response (eye/hand), with all four possible combinations (manual short; manual long; saccadic short; saccadic long) varying on a trial-by-trial basis.
4. In the ‘Neutral’ condition, the cue provided neither temporal nor motor information: both the time between cue and target, and the motor effector to be used to respond, were determined only upon target presentation, with all four possible combinations varying on a trial-by-trial basis.

Each of the four experimental conditions was presented in a block of 93 trials (80 target trials and 13 catch trials). In catch trials (14% of the total number of trials), the cues were not followed by a target event and subjects were required to maintain fixation at the centre of the screen without producing a response. Catch trials were included to minimise anticipatory responding and to normalise the differential benefits of temporal orienting at short versus long delays (Correa et al., 2006). Presentation order of the four different conditions was counterbalanced across subjects. Each session lasted less than one hour, and comprised a familiarisation session, installation and calibration of the eyetracker, and approximately 30 min of experimental testing.

#### Analyses

Response RT refers to the time between target onset and the onset of the saccade or button-press. Accuracy refers to the percentage of correct responses. Responses were considered correct when produced a) with the required effector (eye OR hand) and b) toward the required direction (right/left). RTs faster than 120 ms (2% of total responses) were considered as anticipatory and were therefore omitted from the analysis, as were RTs slower than 1000 ms. Percentage correct and mean RTs for saccadic and manual responses were analyzed separately using ANOVAs with motor cueing (M+/M−), temporal cueing (T+/T−), temporal delay (750 ms/1500 ms) and response effector (hand/eye) as repeated-measures factors. The motor and temporal cueing factors constituted the main effects in the 2 × 2 experimental design described above, such that M+ comprised the Motor and Temporal&Motor conditions; M− comprised the Temporal and Neutral conditions; T + comprised the Temporal and Temporal&Motor conditions; and T− comprised the Motor and Neutral conditions. All analyses carried out on mean RTs were replicated with median RTs and the two sets of analyses produced a similar pattern of results. Significant effects revealed by ANOVAs were submitted to post-hoc breakdown analyses (Newman–Keuls post-hoc tests). Statistical threshold was fixed at P < 0.05 for all analyses.

### fMRI experiment

The task used in the fMRI experiment was largely similar to that used in the behavioural experiment (see above for details), except that trials were presented in shorter, more numerous experimental blocks in order to optimise fMRI data acquisition.

#### Subjects

We examined 14 right-handed healthy volunteers (mean age: 24 years; 5 males) with no record of neurological or psychiatric disorders and normal or corrected-to-normal vision. All subjects gave informed written consent to the study protocol, which had been approved by the Oxfordshire Local Research Ethics Committee.

#### Experimental protocol

Subjects performed the same four cueing conditions described for the behavioural experiment. However, in order to maximize the number of useable data points, there were no catch trials in the fMRI session. Furthermore, instead of grouping each cueing condition into a discrete block of 93 trials, trials were grouped into 16 short (~ 35 s) mini-blocks of 10 trials each. Each mini-block began with a brief (2 s) static instruction screen, comprising the name of the cueing condition and a visual reminder of its associated cue. A trial began with brief (100 ms) presentation of a central cue, followed by a delay (ISI: 750 ms/1500 ms), then brief (100 ms) presentation of a central target (vertical/horizontal bar). Average trial length was 1395 ms (range = 1020 ms–1770 ms). Trials were separated by a variable (1500 ms–3300 ms) inter-trial interval to ensure random sub-sampling of the brain volume relative to each of the trial-types. The motor effector used to produce the response (eye/hand) and the time between cue and target presentation (750 ms/1500 ms) varied randomly on a trial-by-trial basis to ensure optimisation of event-related signal strength (Josephs and Henson, 1999).

Two mini-blocks of the same cueing condition were presented successively, separated by one shorter block (~ 16 s, range = 13.7–18.3 s) of a central fixation baseline condition, during which no visual event occurred. This “sandwich” structure was chosen to optimise both the block-design aspect of the fMRI analysis and the attentional set of the subject. By inserting a baseline block between the two cueing mini-blocks, the attentional set of the subject is simply put ‘on-hold’ rather than switching to a new cueing condition, and each cueing mini-block lasts only 35 s thus avoiding being filtered out as a source of low-frequency drift. Each of the four cueing blocks (each block comprising 2 mini-blocks and the intervening baseline block) were presented in pseudo-randomised permuted order, with the proviso that the same cueing block could not be presented twice in a row. Presentation order of each cueing block was counterbalanced across subjects. In total, subjects performed 360 trials in a session. An initial familiarization session, performed outside the scanner, ensured subjects had learnt the task.

#### Functional magnetic resonance imaging

Subjects lay supine in a Siemens Trio whole-body scanner (Erlangen, Germany) operating at 3 T and equipped for echo-planar imaging. Each volume of 35 slices (4 mm thickness) was acquired using blood-oxygen-level-dependent (BOLD) contrast (in plane resolution = 3 × 3 mm, TR = 2.11 s, interleaved acquisition, oblique slices 30° to the axial). Visual stimuli projected on a screen placed at the back of the scanner were presented to the subject (LCD projector, 60 Hz) via a mirror system that also allowed eye position monitoring using a remote infra-red eyetracker (Applied Science Laboratories, Bedford, MA; 60 Hz). Magnet-compatible electronic switches attached to a response box were used to record button-presses performed with left or right index fingers (1000 Hz). A structural MRI was also acquired (using a standard T1-weighted scanning sequence, 1 mm^3^ resolution) to allow anatomically specific localization of significant areas of brain activation.

#### Behavioural data analysis

Percentage correct and mean RTs for saccadic and manual responses performed during the scanning session were analyzed separately using ANOVAs, as for the Behavioural Experiment. Significant effects were submitted to post-hoc breakdown analyses (Newman–Keuls post-hoc tests). Statistical threshold was fixed at P < 0.05 for all analyses.

#### fMRI data analysis

Image processing and analysis of fMRI data were conducted with SPM8 (http://www.fil.ion.ucl.ac.uk/spm/software/spm8). 1777 fMRI volumes were acquired for each subject. The first 5 images allowed for magnetic field saturation and were discarded. All remaining functional images were slice-time corrected using the middle slice in time as reference (slice #33; 35 slices total; interleaved acquisition). After discarding the last two volumes, these images were then realigned to correct for head movement between scans. Each structural MRI was co-registered to the corresponding mean realigned functional image, in order to put structural images into the functional brain space. All functional images were then spatially normalised by matching each image to the standard SPM8 EPI template, resampled to 3-mm isotropic voxel size, and were spatially smoothed using isotropic Gaussian kernels of 8 mm full-width half-maximum (FWHM). Stimulus-evoked neural responses were modelled as single events that were time-locked to the onset of the cue and then convolved with a canonical haemodynamic response function (HRF). Data were analyzed for regionally specific changes in HRF amplitude.

We modelled 16 regressors, comprising the factorial combination of the 4 cueing conditions (Motor, Temporal, Temporal&Motor, Neutral), the 2 response effectors (hand, eye), and the 2 delays (750 ms/15000 ms) (i.e., MotorHand750ms, MotorHand1500ms, MotorEye750ms, MotorEye1500ms, TemporalHand750ms… etc.). Experimental effects were estimated according to the general linear model at each voxel in brain space in each of the 14 subjects. Images were adjusted for low-frequency physiological drifts, using a high-pass filter of 128 s. At the first level of analysis, 14 separate single-subject analyses were performed for each contrast of interest. Contrast images from these individual statistical parametric maps (SPM) of the t statistic (transformed into corresponding Z values) were entered into a second level analysis to derive statistical inferences using one-sample t tests.

Primary contrasts of interest identified regions associated with temporal orienting in the absence of motor predictability, or with motor orienting in the absence of temporal predictability. To index temporal orienting, we compared trials in which the time of target onset, but not the motor effector for response, could be predicted to trials in which neither could be predicted (i.e., Temporal – Neutral). Similarly, to measure motor orienting we compared trials in which information concerning the motor effector, but not the time of target onset, was provided to trials in which neither source of information was provided (i.e., Motor – Neutral). Finally, to assess the interaction between temporal and motor orienting, we compared trials in which both temporal and motor information was provided to trials in which only one of these sources of information was provided (i.e., [Time&Motor – Time] – [Motor-Neutral]).

These contrasts averaged cue-specific responses across short and long delays, and across manual and saccadic response effectors. However, one of the aims of this study was to explore temporal orienting during saccadic response trials and to compare its neural correlates to those of temporal orienting during manual trials. Any overlap in temporal orienting areas during manual versus saccadic trials would provide evidence for a centralised, effector-independent mechanism for temporal orienting. Therefore, to evaluate the simple main effects of cue-type separately for manual and for saccadic responses we further sub-divided the Motor and Temporal orienting contrasts by effector-type (i.e., [TemporalHand–NeutralHand]; [TemporalEye–NeutralEye]; [MotorHand–NeutralHand]; [MotorEye–NeutralEye]).

As well as exploring each of these four contrasts individually, we also searched for areas that were common to pairs of contrasts. We first looked for temporal (or motor) orienting regions that were common to both manual and saccadic response trials. Effector-independent temporal orienting regions were identified by inclusively masking [TemporalHand–NeutralHand] with [TemporalEye–NeutralEye], and vice versa (both maps thresholded at p < 0.001, uncorrected). Similarly, effector-independent motor orienting regions were identified by masking [MotorHand–NeutralHand] with [MotorEye–NeutralEye], and vice versa. We then looked for manual- (or saccade-) specific regions that were common to temporal and motor orienting by inclusively masking [TemporalHand–NeutralHand] with [MotorHand–NeutralHand] (or [TemporalEye–NeutralEye] with [MotorEye–NeutralEye]). The non-orthogonality of these latter contrasts (in which the NeutralHand (or NeutralEye) condition is shared across both contrasts) guided our decision to examine commonalities using the more conservative inclusive masking procedure rather than conjunction analysis (Nichols et al., 2005). However, it is worth noting that all inclusive masking results were replicated by equivalent conjunction analyses.

Finally, in order to identify effector-specific temporal and motor orienting regions, we directly contrasted manual and saccadic trials against one another. For example, temporal orienting regions specific to saccadic or manual responses were identified by the interaction terms ([TemporalEye–NeutralEye]–[TemporalHand–NeutralHand]) and ([TemporalHand–NeutralHand]–[TemporalEye–NeutralEye]) respectively. Equivalent interactions were interrogated for the Motor orienting condition.

The resulting activations were characterized in terms of both peak amplitude and spatial extent. The significance of each activation was estimated using distributional approximations from the theory of Gaussian fields. In regions for which we had strong *a priori* hypotheses based on previous studies, we adopted a significance threshold that was uncorrected for multiple comparisons (p < 0.001). These regions comprised left parietal and left premotor cortices (Coull and Nobre, 1998; Rushworth et al., 2003). These regions were further subject to a small volume correction procedure, thresholded at p < 0.05 corrected for Family Wise Error (FWE). We used pre-defined anatomical regions of interest (ROI) from the Marsbar ROI toolbox (Brett et al., 2002) (viz., left parietal (inferior, superior and supramarginal gyrus) cortex and left BA44) to define restricted search volumes. For all other regions, we adopted a significance threshold of p < 0.05 corrected for multiple comparisons (FWE). So as to ensure that reported clusters were due to activations induced by the experimental condition (Temporal or Motor cues), rather than deactivations induced by the control condition (Neutral cue), all statistical maps resulting from the contrasts described previously were inclusively masked by the maps of each experimental condition compared to baseline, thresholded at p < .05, uncorrected for multiple comparisons (e.g., the effect of Temporal orienting [Temporal–Neutral] was masked by the map [Temporal–baseline], thresholded at p < .05). The maps of each experimental condition compared to baseline are illustrated in the Supplementary Material. Finally, parameter estimates (beta values) in clusters of theoretical interest were extracted using the Marsbar ROI toolbox and were then plotted to aid data interpretation.

## Results

### Behavioural experiment

**Fig. 2:**
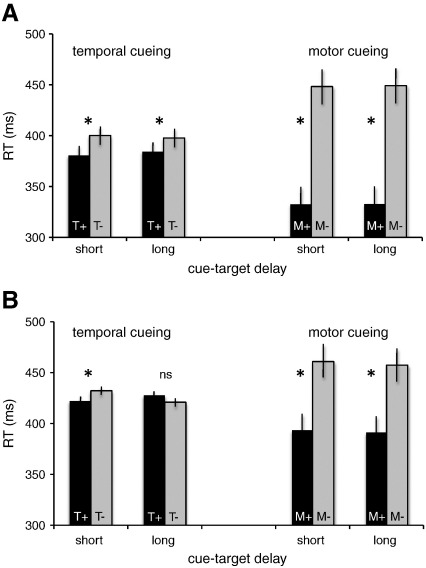
Mean reaction times (RTs) for (A) the behavioural pilot session and (B) the fMRI session, as a function of the type of advance information delivered by the cues (M: motor effector information, T: temporal information; + = information present, − = information absent) and the delay between the cue and target (short = 750 ms, long = 1500 ms). Statistically significant effects are indicated by an asterisk; ns = non-significant.

Mean reaction times (RTs) were analyzed using ANOVAs with temporal cueing (T+/T−), motor cueing (M+/M−), response effector (hand/eye) and temporal delay (750 ms/1500 ms) as repeated-measures factors. A significant main effect of temporal cueing confirmed that temporal orienting speeded RTs (F(1,14) = 13.84, p < .005). There was no significant interaction between temporal cueing and effector (F(1,14) = 0.11, ns) nor between temporal cueing and delay (F(1,14) = 0.78, ns). Temporal orienting therefore speeded RTs equally for saccadic and manual responses, and at both short and long delays (Fig. 2A).

A significant main effect of motor cueing (F(1,14) = 176.42, p < .0001) was qualified by an interaction with response effector (F(1,14) = 5.58, p < 0.05) revealing that motor cues benefitted manual responses (a benefit of 131 ms) more than saccadic ones (a benefit of 101 ms). There was no significant interaction between motor cueing and temporal delay (F(1,14) = 0.01, ns). A significant main effect of response effector (F(1,14) = 25.83, p < 0.0005; mean saccadic RT = 344 ms, mean manual RT = 438 ms) was qualified by an interaction with temporal delay (F(1,14) = 15.57, p < 0.005) demonstrating that RTs were faster (by 11 ms) at long versus short delays for saccadic RTs, but faster at short delays (by 12 ms) for manual RTs.

There was no significant interaction between temporal and motor cueing (F(1,14) = 1.77, ns), revealing that combining motor with temporal cues did not improve performance beyond the benefits of either cue-type alone. All other effects and interactions were non-significant. In summary, these results confirm that participants could reliably orient attention independently toward either a motor effector or a specific moment in time in order to speed performance.

Across conditions, the vast majority (90%) of trials were performed correctly. However, a significant main effect of motor orienting (F(1,14) = 48.29, p < .0001) revealed that responses were more accurate when subjects could prepare the motor effector to be used (93%) than when they could not (86%). And a significant main effect of response effector (F(1,14) = 16.85, p = 0.001) showed that responses were more accurate for saccadic responses (93%) than manual ones (86%). No other main effects or interactions reached significance.

### fMRI experiment

#### Behaviour

In line with the results of the behavioural experiment, analysis of behavioural data collected during the fMRI session showed benefits of both motor (F(1,12) = 67.78, p < 0.0001) and temporal cueing, although the temporal cueing effect interacted with delay (F(1,12) = 15.85, p < 0.005). Post-hoc tests revealed that the temporal cueing benefit was significant only at the short, not long, delays (Fig. 2B). This asymmetric cueing benefit is consistent with previous studies (Coull and Nobre, 1998; Coull et al., 2000) and is due to changing conditional probabilities of target appearance as a function of elapsing time (the “hazard function”, Niemi and Näätänen, 1981): in neutral trials, when the target does not appear at the short delay it must, by process of elimination, appear at the longer one, thus effectively removing the cost of the neutral cue. The lack of interaction between temporal cueing and delay in the behavioural study (see above) is likely due to the inclusion of catch trials in that study (Correa et al., 2006) as they add a degree of uncertainty at the longer delay. We chose not to include catch trials in the fMRI session so as to maximize the number of useable data points in the limited scanning time available. Although this experimental strategy admitted the possibility of a hazard function effect during neutral trials, we hypothesised that the hazard function would play a minimal role during the crucial temporal cueing trials since temporal cues predicted with 100% certainty when the target would appear. Post-hoc tests showed that RTs did not differ between short and long delays in the temporal cueing condition, thus confirming this hypothesis.

A significant main effect of response effector (F(1,12) = 124.34, p < 0.0001; mean saccadic RT = 347 ms, mean manual RT = 505 ms) was qualified by an interaction with temporal delay (F(1,12) = 11.71, p = 0.005) demonstrating that although RTs were faster (by 11 ms) at long versus short delays for saccadic RTs, manual RTs did not differ across delays. As in the behavioural experiment, motor and temporal orienting did not interact (F(1,12) = 2.17, ns). All other interactions were non-significant. Taken as a whole, these results confirm that during the fMRI session subjects used temporal and motor cues to orient attention towards the predicted onset time, or response effector, respectively.

Across conditions, a large majority (90%) of trials were performed correctly. However, a significant main effect of motor orienting (F(1,12) = 8.35, p < .05) revealed that responses were more accurate when subjects could prepare the motor effector to be used (91%) than when they could not (88%) while a significant main effect of response effector (F(1,12) = 14.69, p < 0.005) showed that responses were more accurate for saccadic responses (91%) than manual ones (88%). No other main effects or interactions reached significance.

### Neuroimaging

#### General response preparation networks

Compared to baseline, all four experimental conditions activated largely overlapping regions of frontal, parietal and occipital cortices, as would be expected for performance of a visual choice-RT task (Supplementary Material). Planned contrasts (see below) identified differential areas of activation within these networks, depending on whether temporal or motor effector information was used to predict when or how the response would be executed.

#### Temporal orienting

Based on previous fMRI studies assessing neural substrates of temporal orienting (Coull et al., 2001; Coull and Nobre, 1998), we predicted selective activation of left inferior parietal and left premotor regions when subjects could predict the moment at which the target would be presented. Indeed, the effect of temporal orienting, averaged across response effectors [Temporal – Neutral], revealed significant activity (p < 0.05, corrected for multiple comparisons using Family Wise Error) in a large left-lateralized parietal network (Table 1a). These activations centred around left intraparietal sulcus (IPS), extending into left inferior and superior parietal cortices. We found no evidence for activation of left ventral premotor cortex. Since previous studies (Coull et al., 2000; Coull and Nobre, 1998) reporting premotor activations employed manual responses only, it is possible that premotor activations are absent when subjects do not have a fixed manual response-set but must switch from manual to ocular responses on a trial-by-trial basis. The analysis of the main effects of temporal orienting [(Temporal + Temporal&Motor)-(Motor + Neutral)] confirmed the results of this simple main effects analysis, with one cluster in left IPS (peak at − 27, − 69, 51, Z = 5.44) and another in left supramarginal gyrus (peak at − 45, − 39, 54, Z = 5.00).

**Fig. 3:**
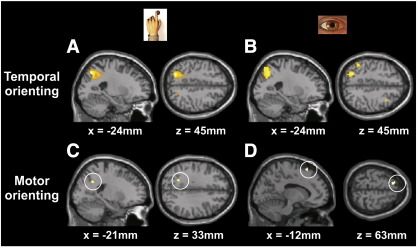
Temporal orienting invoked activations in left intraparietal sulcus for both A) manual and B) saccadic response trials. Motor orienting invoked activation C) deep in left intraparietal sulcus for manual responses and D) in left preSMA for saccadic eye responses. Activations are displayed on a standard SPM template brain. Spatial co-ordinates (mm) define the anatomical location of each slice. Activations are thresholded at p < 0.001, uncorrected for multiple comparisons.

**Fig. 4:**
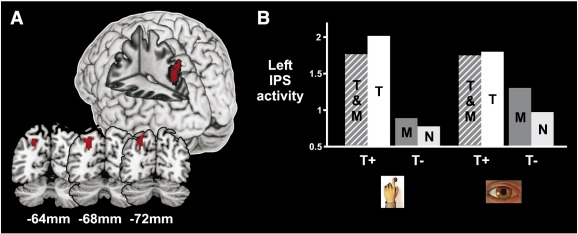
The only cluster of activation common to both manual and saccadic temporal orienting trials lies in left intraparietal sulcus. (A) This activation is displayed on a standard template brain using MRIcron software (www.mricro.com/mricron). Spatial co-ordinates (mm) define the anatomical location of each slice in the y-dimension. Activations are thresholded at p < 0.001, uncorrected for multiple comparisons. The left hemisphere is on the left side of the figure. (B) Mean level of activity in this intraparietal sulcus cluster for each of the four cueing conditions (T&M:Temporal&Motor; T: Temporal; M: Motor; N: Neutral) during either manual or saccadic response trials. Compared to trials in which no temporal information is available (T−), temporal cueing (T+) activates left intraparietal sulcus whether the effector that will be used to register the response can be prepared in advance (T&M) or not (T).

To examine the possibility that distinct, effector-specific, networks may underlie temporal orienting, the [Temporal–Neutral] contrast was examined for trials in which responses had been registered with manual responses separately from those in which responses were saccadic. Temporal orienting activated left-lateralized parietal cortex both for manual and for saccadic responses (Table 1b; Figs. 3A and B). Inclusively masking temporal orienting during saccadic trials with temporal orienting during manual trials revealed a large cluster of common activity in left intraparietal sulcus (Table 1c; Fig. 4). To identify effector-specific temporal orienting areas we directly compared temporal orienting during saccadic trials to temporal orienting during manual trials. Temporal orienting in saccadic trials invoked selective activation of left supramarginal gyrus (Table 1b; the transverse section of Fig. 3B illustrates its spatial discontiguity with the intraparietal sulcus cluster). By contrast, temporal orienting in manual trials did not selectively activate any brain areas over and above those activated during saccadic trials, even at an uncorrected threshold. In summary, the neural network involved in temporal orienting principally comprises left parietal cortex, centred around the intraparietal sulcus, with this area being engaged whether temporal orienting is eventually followed by a saccadic or a manual response.

#### Motor orienting

Based on previous data (Rushworth et al., 2003), we predicted activation of left parietal regions (supramarginal gyrus and intraparietal sulcus) when subjects could selectively prepare the motor effector that would be required to produce the response. Motor orienting [Motor-Neutral] showed no significant areas of activation, even at a liberal threshold (p < .01 uncorrected for multiple comparisons). The main effect of motor orienting [(Motor + Temporal&Motor)–(Temporal + Neutral)] similarly failed to show any areas of significant activation. However, these contrasts were averaged across response effectors. Given the largely segregated nature of the manual and oculomotor systems, this null result is not particularly surprising. We therefore examined the [Motor-Neutral] contrast for manual and saccadic responses separately. The simple main effect of motor orienting for manual responses [MotorHand–NeutralHand] revealed activation deep in left intraparietal sulcus (Fig. 3C; peak at − 21 − 54 33 mm, z = 3.80; p < 0.001 uncorrected for multiple comparisons). By contrast, the simple main effect of motor orienting for saccadic responses [MotorEye–NeutralEye] revealed a single cluster of activation in left preSMA, anterior to the Supplementary Eye Fields (Fig. 3D; peak at − 12 18 63 mm, z = 3.70; p < 0.001 uncorrected for multiple comparisons). Although we had no *a priori* hypothesis that preSMA would be activated by saccadic motor orienting, we have nevertheless decided to report it given 1) preSMA's involvement in motor preparation processes in general (Cunnington et al., 2003; Curtis and D'Esposito, 2003; Lee et al., 1999) and 2) the role of rostral preSMA in resolving saccadic response competition (Nachev et al., 2005). Interaction analyses directly comparing saccadic versus manual motor orienting revealed no significant areas of activation. However, it is of note that, in contrast to manual motor orienting, saccadic motor orienting did not activate left parietal cortex, even at a more liberal threshold (p < 0.01, uncorrected for multiple comparisons). Inclusively masking manual motor orienting with saccadic motor orienting confirmed that there were no areas of common activity across response effectors. In summary, results show that motor orienting engaged anatomically distinct regions during saccadic versus manual response preparation, although the direct comparison of these two contrasts failed to show effector-specific regions.

#### Motor and temporal orienting commonalities

Activations common to both motor and temporal orienting were identified by inclusive masking procedures. Masking the simple main effect of temporal orienting during manual trials by the simple main effect of motor orienting during manual trials (or vice versa) revealed an activation common to temporal and motor orienting deep in left IPS (Fig. 3C; peak at − 21 − 54 33 mm, z = 3.80; p < 0.001 uncorrected for multiple comparisons). By contrast, performing an analogous inclusive masking procedure for saccadic trials revealed no areas of common activity (even at a liberal threshold, p < .01 uncorrected for multiple comparisons).

#### Motor × Temporal orienting interaction

The interaction between Motor and Temporal orienting [(Temporal&Motor–Motor) – (Temporal–Neutral)] failed to reveal any areas of significant activation, whether conditions were averaged across response effectors or assessed separately for saccadic and manual responses. The more straightforward comparison of Temporal&Motor to the Temporal and Motor conditions [Temporal&Motor – (Temporal + Motor)] also failed to show any significant areas of activation. This lack of neural interaction parallels the lack of behavioural interaction between temporal and motor orienting, noted above.

## Discussion

### Temporal and motor orienting are functionally discrete attentional processes

We used a fully factorial experimental design to disambiguate the functional consequences and neural systems mediating temporal and motor orienting. Specifically, a choice-RT task employed symbolic pre-cues to inform subjects as to when (short/long delay) and/or with which motor effector (oculomotor saccade/index finger button-press) a speeded response to a subsequent target would be required. Behaviourally, temporal cues speeded responding as compared to neutral cues, even when the motor effector used to register the response was unpredictable. This confirms that temporal orienting can benefit performance independently from motor orienting and, therefore, that time is a useful stimulus parameter for optimising behaviour. Moreover, behavioural benefits were observed whether responses were registered with saccades or button-presses. This result extends and clarifies prior reports of temporal orienting paradigms that used only manual responses to measure performance (Coull and Nobre, 1998; Griffin et al., 2001). Previous behavioural studies have shown speeding of smooth pursuit eye movements with symbolic temporal cues (Badler and Heinen, 2006; Jarrett and Barnes, 2005), or of saccadic eye movements with a more exogenous manipulation of temporal predictability of inter-stimulus intervals (Bronstein and Kennard, 1987), but this is the first study to show improved performance on saccadic onset times with symbolic cues.

We not only provide evidence that temporal and motor effector cues speed performance independently, we also show that they do not interact with one another. Knowing both how *and* when to execute the response did not improve performance to any greater extent than simply knowing *how* to execute it, which itself improved performance to a significantly greater extent than knowing *when* to execute it. These behavioural results highlight the primacy of motor effector, rather than temporal, information in response preparation. Second, they confirm that the observed effects are not due simply to variations in the number of response possibilities but, rather, reflect differences in selectively preparing discrete components of the response. If informative cues were speeding RTs due to a reduction in response uncertainty then (1) RT benefits should be greatest when response uncertainty is at its lowest, i.e., during the combined Temporal&Motor condition. This is not the case. (2) RT benefits should be equivalent for Temporal and Motor conditions in which response uncertainty was matched. Again, this is not the case. Although it may be argued that the Motor condition contained implicit temporal information (omission of the target at the short delay guaranteed its appearance at the longer delay), RTs in the Motor condition were equally fast at both short and long delays, suggesting this implicit form of temporal preparation had minimal impact on resulting behaviour.

### Effector-independent substrate for temporal orienting in left intraparietal sulcus

Temporal, compared to neutral, cueing selectively activated left intraparietal sulcus (IPS), thus confirming previous reports using this paradigm (Coull and Nobre, 1998; Coull et al., 2000; Coull et al., 2001). Notably, left-sided lateralisation persisted despite the fact that subjects responded equally with left or right-sided movements. Left-lateralised parietal activation is also consistent with the results of previous collision judgment studies (Assmus et al., 2003, 2005; Coull et al., 2008) in which the dynamics of stimulus motion allowed subjects to predict the time at which a moving stimulus would reach a certain location. Despite the spatial component of these studies, as well as the fact that subjects were making perceptual judgments, and not speeded motor responses as in our temporal orienting paradigm, the inherent temporal predictability of stimulus motion selectively activated *left* parietal cortex. By contrast, other studies have linked temporal predictability to right-sided prefrontal and parietal cortices (Vallesi et al., 2007, 2009; Bueti et al., 2010). However, these studies measured how temporal predictability evolves as a function of the passage of time itself (the “hazard function”) rather than whether temporally predictable information is available in the first place (e.g. via temporal cues) or not (e.g. neutral cues). Yet Triviño et al. (2010) have more recently reported that temporal orienting is also compromised in patients with right prefrontal cortex lesions. So why do we fail to see right prefrontal cortex in our fMRI study? One explanation is that right prefrontal cortex is activated not only by the temporal cue but also by the neutral cue. In the neutral condition, subjects can use the hazard function to reorient attention to the later time-point as soon as the cue has failed to appear at the earlier time-point. A direct comparison of the temporal to the neutral cue would therefore effectively subtract out right prefrontal cortex activation. Considered as a whole, these data suggest that right-sided cortical areas are critical for updating temporal predictions as a function of time-in-passing, whereas left parietal cortex is engaged when a fixed temporal prediction is deployed in the first place.

Moreover, our factorial design revealed, for the first time, that this activation was independent from potentially coincident motor preparation processes. Our previous studies (e.g., Coull and Nobre, 1998) measured temporal orienting for a single (manual) response effector, giving subjects the opportunity to prepare not only the time, but also the hand, of response. Incidental motor orienting is an unlikely explanation for our previous results however since response preparation requirements were matched across tasks. Moreover, the fully factorial design of the present experiment, in which temporal and motor components of response preparation were cued independently, confirmed unambiguously that temporal cues activate left IPS even when the response effector that will be used to register the response is, as yet, unknown. Temporal cues activated the same region of left IPS whether the eventual motor response constituted a manual button-press or an oculomotor saccade. In other words, left IPS represents an effector-independent substrate for temporal orienting. Temporal orienting therefore appears to function as an attentional mechanism that operates with similar principles on the ocular or manual control systems. Spatial orienting has previously been shown to modulate the neural substrates of oculomotor or manual behaviour in similar ways (Astafiev et al., 2003; Eimer et al., 2005). Our data provide a temporal analogue to these spatial studies.

The region of parietal cortex particularly implicated in temporal orienting is the left IPS and adjacent supramarginal gyrus. The left hemisphere plays a dominant role in action selection, particularly in feedforward aspects of motor control (Serrien et al., 2006), while IPS is heavily implicated in reaching, pointing and grasping movements of the hand (Culham and Valyear, 2006). Fittingly therefore, left IPS is precisely the area we have also found to be activated by motor orienting to *hand* movements in particular (see also Rushworth et al., 2001) and has previously been implicated in both motor preparation (Krams et al., 1998) and motor intention (Lau et al., 2004) for hand movements. Neuroanatomical overlap between temporal orienting and manual motor orienting is reminiscent of the neural overlap between spatial orienting and oculomotor preparation predicted by the premotor theory of attention (Rizzolatti et al., 1994; although see Liu et al. (2010) for recent evidence of functional dissociation within discrete regions of intraparietal cortex). In its simplest form, the premotor theory of attention suggests that spatial orienting is a functional corollary of oculomotor preparation. By analogy therefore, one could hypothesise that temporal orienting could be conceptualized as a corollary of manual motor preparation. However, our observations that temporal orienting is functionally dissociable from motor orienting, that it speeds saccadic, as well as manual, responses, and that the neural substrates of temporal orienting are effector-independent refute this hypothesis, and instead suggest that temporal orienting is not merely a causal effect of hand movement preparation.

So if temporal and manual motor orienting are behaviourally dissociable, what is the functional significance of their (partial) neuroanatomical overlap? One possibility is that manual motor areas are being recruited, or “neurally recycled”, for temporal prediction in much the same way that, for example, spatial areas are recruited for number processing (Dehaene and Cohen, 2007). In everyday situations, anticipating *when* an action will take place may be associated more often with preparation of limb movements (see also O'Reilly et al., 2008), for example catching a ball or hitting the brake before the traffic light turns red, than of saccadic eye movements. The same brain area may therefore become functionally specialized for these distinct, yet often coincident, processes.

### Effector-dependent neural substrates for motor orienting

Rushworth et al. (2001) concluded that, in contrast to the oculomotor spatial attention system of the right parietal cortex, left parietal cortex embodied a *manual* motor attention system. Our event-related fMRI data confirm this conclusion by demonstrating that, as compared to neutral cue trials, left IPS was activated by motor orienting to hand movements but not by motor orienting to eye movements. Our data further reveal a distinct neural basis for oculomotor orienting in an area of preSupplementary Motor Area (preSMA) that lies anterior to the Supplementary Eye Fields. preSMA has previously been linked to voluntary saccade control (Curtis and D'Esposito, 2003; Nachev et al., 2005) and, more specifically, to postponement of saccade onset (Boxer et al., 2006; Yang et al., 2008). These data, along with our own, indicate a specific role for preSMA in non-spatially selective preparation of the oculomotor system in general. More generally, the dissociable pattern of results for eye and hand motor orienting reflect the existence of effector-dependent neural substrates for motor orienting.

### Dissociating motor orienting for hand and eye movements from spatial orienting

We have shown attentional benefits of motor effector cues on eye, as well as hand, movements. Eye movements have previously been shown to share neural substrates with spatial attention (Corbetta et al., 1998; Moore and Fallah, 2001; Nobre et al., 2000), a result that is taken as evidence for shared functional substrates and, more specifically, for the premotor theory of attention (Rizzolatti et al., 1994). According to these views, saccadic eye movements are functionally coincident with the spatial orienting of attention. Could the benefits of motor effector cues on saccade speed in our own data therefore be due to spatial, rather than motor, orienting effects? Moreover, given that shifts in spatial attention have been associated not only with saccade preparation (Deubel and Schneider, 1996; see Awh et al., 2006 for a review) but also with preparation of spatially discrete manual responses (Eimer et al., 2005, 2006), the observed motor orienting benefits on manual responses may also be due to spatial orienting effects. Critically, however, we asked subjects to select and prepare an entire effector system (manual/ocular) rather than spatially specified components within that effector system (e.g., left/right manual response), therefore confirming that saccadic oculomotor preparation can occur in the absence of spatial orienting. Although prior fMRI studies have also investigated the neural correlates of non-spatial saccade preparation (Brown et al., 2007; Curtis and Connolly, 2008), these paradigms manipulated only whether the spatial location of the planned saccade was known in advance or not. Here, by contrast, we show that attention can be selectively directed between-effectors, allowing subjects to prepare the oculomotor system in favor of another distinct effector system (here, the manual response system).

The fact that our paradigm entails a non-spatial form of oculomotor preparation may also be crucial in interpreting the lack of parietal activation for motor orienting to eye movements. This result may seem surprising given that many electrophysiological and imaging studies have already implicated the lateral intraparietal area (LIP) in oculomotor preparation (see Grefkes and Fink, 2005 for a review), even when the spatial location of the planned movement is unknown (Dickinson et al., 2003). However, a direct comparison of spatial to non-spatial oculomotor preparation shows that LIP is engaged *particularly* when the spatial location of the planned saccade is known in advance (Curtis and Connolly, 2008). In this case, our finding that non-spatial oculomotor orienting does not preferentially activate parietal cortex any more than a neutral cue condition becomes less surprising.

## Conclusions

Prior studies indicate a neural overlap in left parietal cortex for temporal and motor orienting, which could suggest shared functional substrates. However, by careful experimental design, we provide evidence that temporal and motor orienting are functionally dissociable processes, each capable of improving performance independently. Moreover, our fMRI results reveal that temporal orienting activates left IPS regardless of the motor effector used to register the response or the laterality of the movement. We therefore suggest temporal orienting to be an effector-independent attentional mechanism that relies critically upon the functioning of left IPS, thus providing a temporal analogue to the more right-lateralised processes of spatial orienting.

The following are the supplementary materials related to this article.

Supplementary Figure Activations invoked by temporal and motor orienting, for hand and eye responses, as compared to baseline. The baseline condition consisted of static visual fixation and required no motor response. Comparisons between each orienting condition to a well-matched Neutral cue condition are reported in the Results section. Activations are rendered onto lateral and dorsal views of a standard brain template.

## Figures and Tables

**Table 1:** Brain regions recruited by temporal orienting. Activations were assessed either by (a) averaging across response effectors, (b) assessing hand and eye movement trials separately or (c) assessing areas that were common to eye and hand movements. L = left. Z scores are significant at p < 0.001 (uncorrected for multiple comparisons) in the hypothesised region of left parietal cortex. In addition, all activations were significant at a level of p < 0.05 (FWE) when small volume corrections were applied using anatomically pre-defined ROIs. Asterisks indicate activations that also survive a more stringent whole brain correction (FWE) for multiple comparisons at a level of **p < .01 or *p < .05.

Brain areas	x y z co-ordinates (mm)	Z score
(a) Temporal orienting		
Temporal–Neutral		
L intraparietal sulcus	− 24 − 69 45	5.14**
(b) Effector-specific temporal orienting		
*Hand*		
TemporalHand–NeutralHand		
L intraparietal sulcus	− 24 − 69 45	4.86*
**[**TemporalHand–NeutralHand]–[TemporalEye–NeutralEye]		
–	–	–
*Eye*		
TemporalEye–NeutralEye		
L supramarginal gyrus	− 51 − 51 33	4.32
L intraparietal sulcus	− 21 − 72 54	4.00
**[**TemporalEye–NeutralEye]–[TemporalHand–NeutralHand]		
L supramarginal gyrus	− 51 − 66 30	3.50
(c) Effector-independent temporal orienting		
[TemporalHand–NeutralHand] & [TemporalEye–NeutralEye]		
L intraparietal sulcus	− 21 − 72 54	4.00
