# Supplementary material for: Functionally dissociating temporal and motor components of response preparation in left intraparietal sulcus
Source: Neuroimage. 2011 Jan 15;54(2-3):1221–30. doi: 10.1016/j.neuroimage.2010.09.038 (PMC3025354; doi:10.1016/j.neuroimage.2010.09.038)
Supplement: Supplementary Figure — Activations invoked by temporal and motor orienting, for hand and eye responses, as compared to baseline. The baseline condition consisted of static visual fixation and required no motor response. Comparisons between each orienting condition to a well-matched Neutral cue condition are reported in the Results section. Activations are rendered onto lateral and dorsal views of a standard brain template. [file mmc1.pdf]

Supplementary Material

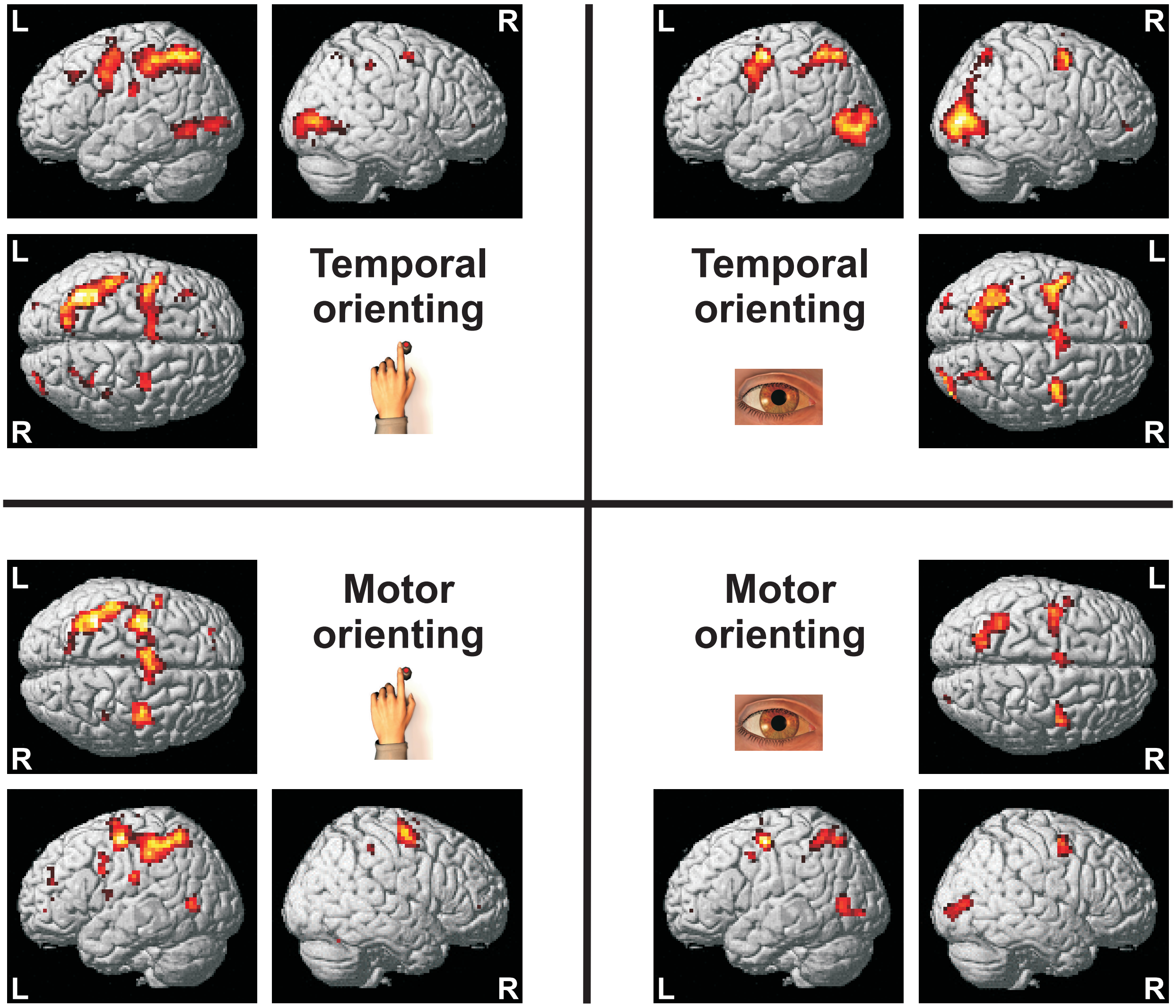

Supplementary Figure. Activations invoked by temporal and motor orienting, for hand and eye responses as compared to baseline. The baseline condition consisted of a static visual fixation, and required no motor response. Comparisons between each of these conditions to a well-matched Neutral cue condition are reported in the Results section. The activations are rendered onto lateral and dorsal views of a standard brain template.
